# Physicochemical evaluation and essential oil composition analysis of **Hyssopus cuspidatus** Boriss from Xinjiang, China

**DOI:** 10.4103/0973-1296.71790

**Published:** 2010

**Authors:** Xiaoying Zhou, Gong Hai-Yan, Xu Tun-Hai, Shuge Tian

**Affiliations:** 1 *Xinjiang Key Laboratory of Famous Prescription and Science of Formulas, China*; 2 *College of Pharmacy, China*; 3 *School of Traditional Chinese Medicine, Beijing University of Chinese Medicine, Beijing 100102, China*; 4 *College of TCM, XinJiang Medical University, Urumqi 830011, XinJiang, China*

**Keywords:** Essential oil, GC-MS, **Hyssopus cuspidatus** Boriss, pharmacognosy

## Abstract

**Background:**

It is reported that the plant **Hyssopus cuspidatus** Boriss from Xinjiang has great value. This article deals with the detailed pharmacognostic evaluation of the crude drug **H. cuspidatus** Boriss.

**Materials and Methods:**

The essential oil of **H. cuspidatus** Boriss from Xinjiang, China, was extracted by the method of hydrodistillation and the chemical composition of the essential oil was analyzed by gas chromatography-mass spectrometry (GC–MS).

**Results:**

The yield of essential oil based on the dry weight of the plant was 0.6%(w/w). Fifty compounds accounting for 99.42% of the total oil were identified. The major components were oxygenated terpenes (66.33%), monoterpenes (26.14%), oxygenated sesquiterpenes (1.25%), and octane (1.85%).

**Conclusion:**

Oxygenated terpenes were the main group of the compounds. The physicochemical parameters presented in this article may be proposed as parameters to establish the authenticity of **H. cuspidatus** Boriss and can possibly aid pharmacognostic and taxonomic species identification.

## INTRODUCTION

It is commonly known that **Hyssopus cuspidatus** Boriss has various uses in the Xinjiang medicinal system, but there are few high-quality human trials researching these uses. It has been used traditionally as an antispasmodic for its antispasmodic action,[1] expectorant, emmenagogue (stimulates menstruation), stimulant, carminative (digestive aid), peripheral vasodilator, anti-inflammatory and anticatarrhal agent, tonic, and sweat-inducer.

**H. cuspidatus** Boriss and its oil are mainly used in the treatment of respiratory diseases. Hippocrates recommended using hyssop to treat bronchitis. Today, **H. cuspidatus** Boriss is used in the treatment of nasal congestion and mild irritations of the respiratory tract. The essential oil contains pinocamphone and isopinocamphone, which have neurotoxic effects.[2,3]

**H. cuspidatus** Boriss has two native and one cultivated species in China, which is one of the aromatic endemic plants in the Xinjiang Province, of China. This plant, with the common local name of Shenxiangcao or Uygur’s name zufa, has been of interest to Uygur traditional medicine, especially in Arletai. Infusion obtained from the aerial parts of **H. cuspidatus** Boriss is used traditionally for the following purposes: carminative and antispasmodic, in the treatment of cough, bronchitis, colds and chronic catarrh, and also for its tonic effects on the digestive, urinary, nervous, and bronchial systems.[4,5]

The literature survey showed that the evaluation of the physicochemical properties and the essential oil composition of **H. cuspidatus** Boriss *officinalis* have previously been done, but not for **H. cuspidatus** Boriss.

In spite of the numerous medicinal uses attributed to this plant, pharmacognosy information about this plant has not been published. Hence, the present investigation is an attempt in this direction, including the determination of physicochemical constants, the preliminary phytochemical screening, physicochemical analysis, and essential oil composition analysis of the water extract of **H. cuspidatus** Boriss.

## MATERIALS AND METHODS

### Plant materials and reagents

The aerial parts of **H. cuspidatus** Boriss were collected locally from the Arletai mountain of Xinjiang Province, China. Voucher specimens were deposited in the Traditional Chinese Medicine College Museum of Chinese herbal samples of Xinjiang Medical University.

Solvents, namely, petroleum ether, chloroform, ethanol (95%), methanol, formic acid, and reagents, namely, ammonia, iodine, ferric chloride, acetic acid, nitric acid, sulfuric acid, silicowolframic acid, hydrochloric acid, bromocresol green, α-naphthol, ninhydrin, gelatin, and so on, were purchased from Tianjin Fu-Yu Meticulous Chemical Reagent Company, China.

Gas chromatography-mass spectrometry (GC–MS) analyses were carried out by using Shimadzu QP-2010 GC–MS system.

### Preliminary phytochemical screening

The entire plant was ground to obtain a fine powder, which has been put into experiments. Preliminary phytochemical screening was carried out by using standard procedures according to the official method prescribed.[6] To detect the major chemical groups in **H. cuspidatus** Boriss by qualitative chemical tests, the entire plant extract was obtained by using petroleum ether, ethanol (95%), and water.

### Physicochemical analysis

Physicochemical analyses to obtain the percentage of ash values and extractive values were performed according to the official methods prescribed in the book of extraction and abstraction of active ingredient from plant drug.[7] Fluorescence analysis was carried out according to the method of Chase and Pratt[8] and Kokoski *et al*.[9]

### Essential oil composition analysis

The sample (100 g) was steam distilled with a Clevenger-type apparatus for 6 h. The oil was collected and dried over anhydrous sodium sulfate, then stored at 4°C for analysis.

GC–MS analyses were carried out by using a Shimadzu QP-2010 GC–MS system, operated in the EI mode at 70 eV with scanning from 41 to 450 amu at 0.5 s, using a DB-5 (30 m, 0.25 mm, film thickness 0.25 μm) capillary column. The temperature program was 40°C –250°C at a rate of 5°C/min. Injector and transfer line temperatures were 250°C, the ion source temperature was 200°C. Helium was used as the carrier gas, flow rate 1 mL/min. Split ratio was 1:100.

The identification of the components was made by comparison of their retention time with respect to the n-alkane series (C_6_–C_22_) internal standards. The mass spectra and relative retention indices (RI) were compared with those of commercial (NIST 05 and NIST 05 s). Area percentages were obtained from the TIC response without using an internal standard.

## RESULTS AND DISCUSSION

Preliminary phytochemical screening performed on the various extracts disclosed the presence of terpenes in the petroleum ether, amino acid, flavanoids, terpenes, phenolics, tannins, carbohydrates, and organic acid in ethanol; amino acid, flavanoids, phenolics, and tannins, carbohydrates, and organic acid in water. The results are presented in Table 1.

**Table 1:** Preliminary phytochemical screening of the entire plant power of **Hyssopus cuspidatus** Boriss

Extract	Alkaloids	Amino acid	Flavanoids	Terpenes	Phenolics and tannins	Carbohydrates	Organic acid
Petroleum ether	−	−	−	+	−	−	−
Ethanol		+	+	+	+	+	+
water	−	+	+	−	+	+	+

+Denotes the presence of the respective class of compounds

The total ash value is 4.18% (w/w), acid-insoluble ash value is 0.28% (w/w), most of the total ash is soluble in acid, so acid-insoluble ash value is very low. Meanwhile, the water-soluble ash value is 1.56% (w/w), the water-soluble extractive is very high in this plant, extractive value is 11.47% (w/w). But ethanol-soluble extractive value and ether-soluble extractive value is low, 2.50% (w/w) and 1.79% (w/w), respectively. The results are presented in Tables 2 and 3.

**Table 2:** Ash values of the entire plant of **Hyssopus cuspidatus** Boriss (n = 6)

Parameters	Values % (w/w)
Total ash	4.18
Acid-insoluble ash	0.28
Water-soluble ash	1.56

**Table 3:** Extractive values of the entire plant power of **Hyssopus cuspidatus** Boriss (n = 6)

Parameters	Values % (w/w)
Water-soluble extractive	11.47
Ethanol-soluble extractive	2.50
Ether-soluble extractive	1.79

The result of fluorescence analysis of the entire plant power of **H. cuspidatus** Boriss is presented in Table 4, the fluorescence in day light, UV light (254 nm), and UV light (365 nm) are different from each other when different chemical reagents are added. It indicates that there are various chemical groups existing in the plant.

**Table 4:** Fluorescence analysis of the entire plant power of **Hyssopus cuspidatus** Boriss

Treatment	Day light	UV light (254 nm)	UV light (365 nm)
Powder as such Powder + 5% NaOH	Yellowish brown Yellow	Yellowish green Yellowish green	Yellowish green Yellowish green
Powder + 5% NaOH	Yellow	Yellowish green	Yellowish green
Powder + 10% HCl	Yellowish brown	Brown	Dark brown
Powder + ammonia	Light yellow	Yellow	Light yellow
Powder + iodine	Reddish brown	Light green	Blackish brown
Powder + acetic acid	Yellowish brown	Light brown	Orange
Powder + 5% FeCl_3_	Dark green	Greenish brown	Yellowish green
Powder + 10% H_2_SO_4_	Light brown	Brown	Brown
Powder + 10% HNO_3_	Light brown	Light brown	Light yellow

**Figure 1:**
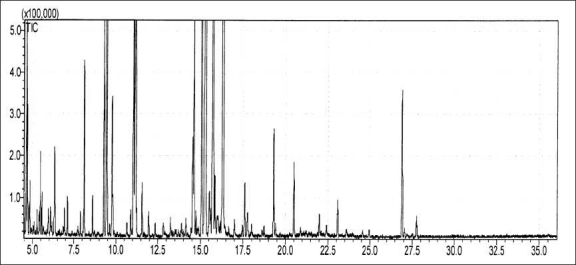
GC—MS chromatogram of essential oil composition of **Hyssopus cuspidatus** Boriss

The air-dried aerial parts of **H. cuspidatus** Boriss were reduced to coarse powder and the oil was isolated by hydrodistillation using a Clevenger-type apparatus for 6 h. The oil was subsequently dried over anhydrous sodium sulfate. The volatile light orange yellow oil (0.6% w/w) was analyzed by GC–MS [Figure 1]. The composition of the essential oil is listed in Table 5 along with the RI of the identified compounds, where all constituents are arranged in such a way that their elution was on the DB-5 column. In total, 38 constituents were identified and quantified, representing 97.89% of the total oil. As can be seen in Table 5, the main constituents identified and their percentage values are as follows: Berbenone (23.84%), β-Pinene (19.76%), Pinocamphone (17.95%), Eucalyptol (7.16%), Myrtenol (7.06%), Methane (3.56%), and l-trans-pinocarveol (3.00%).

**Table 5:** Essential oil composition of **Hyssopus cuspidatus** Boriss

Compound	RI^a^	% Peak area
Cyclopentane, 1-ethyl-2-methyl-, *cis-*	795.8	0.14
Octane	800	1.85
Cyclohexane, 1,2-dimethyl-, trans-	802.9	0.58
Cyclohexane, 1,3-dimethyl-, trans-	808.3	0.26
Heptane, 2,4-dimethyl-	819	0.05
Heptane, 2,6-dimethyl-	826	0.1
Cyclohexane, 1,2-dimethyl-, cis-	830.7	0.18
Cyclohexane, ethyl-	833.9	0.39
Cyclohexane, 1,1,3-trimethyl-	837.7	0.2
Cyclohexane, 1,2,4-trimethyl-	853.3	0.15
Ethylbenzene	858.7	0.19
Octane, 2-methyl-	862.3	0.06
p-Xylene	868	0.66
o-Xylene	892.5	0.14
Nonane	900.4	0.19
α-Thujene	926	0.14
α-Pinene	933.6	0.98
Camphene	950.4	0.21
Sabinen	973.1	1.47
β-Pinene	978.6	19.76
β-Myrcene	989	0.88
2,3-Dehydro-1,8-cineole	990.8	0.21
α-Terpinen	1017.6	0.07
o-Cymene	1025.4	0.15
Limonene	1030	2.11
Eucalyptol	1033.5	7.16
β -trans-Ocimene	1035.7	0.03
β-cis-Ocimene	1046.4	0.33
γ-Terpinen	1058.9	0.16
Isothujol	1071	0.07
α-Campholenal	1128.7	0.11
(1R)-(+)-Norinone	1141	0.65
l-trans-pinocarveol	1143.7	3
*cis*-Verbenol	1147.5	0.17
Methane, [(1-ethynylcyclohexyl)oxy] methoxy-	1158	3.56
Berbenone	1165.2	23.84
α-Terpineol	1172.5	0.34
endo-Borneol	1175.2	0.19
Pinocamphone	1179	17.95
4-Terpineol	1182.8	0.43
Myrtanal	1187.3	0.2
Menthol	1193.6	0.06
Myrtenol	1197.8	7.06
β-Thujone	1240.8	0.4
(+)-Carvone	1246	0.16
Nopol (terpene)	1297.2	0.77
Citronellol acetate	1391.8	0.14
Bicyclo[10.1.0]tridec-1-ene	1431.8	0.27
Palustrol	1578	1.13
Ledol	1613	0.12

a Retention indices relative to C_6_–C_22_ n-alkanes on the DB-_5_ column—preliminary phytochemical screening.
